# Supplementary material for: Fossil Biomarkers and Biosignatures Preserved in Coprolites Reveal Carnivorous Diets in the Carboniferous Mazon Creek Ecosystem
Source: Biology (Basel). 2022 Aug 30;11(9):1289. doi: 10.3390/biology11091289 (PMC9495973; doi:10.3390/biology11091289)
Supplement: Supplementary file 1 [file biology-11-01289-s001.zip › Supplementary Materials Figure S1.pdf]

Supplementary Materials

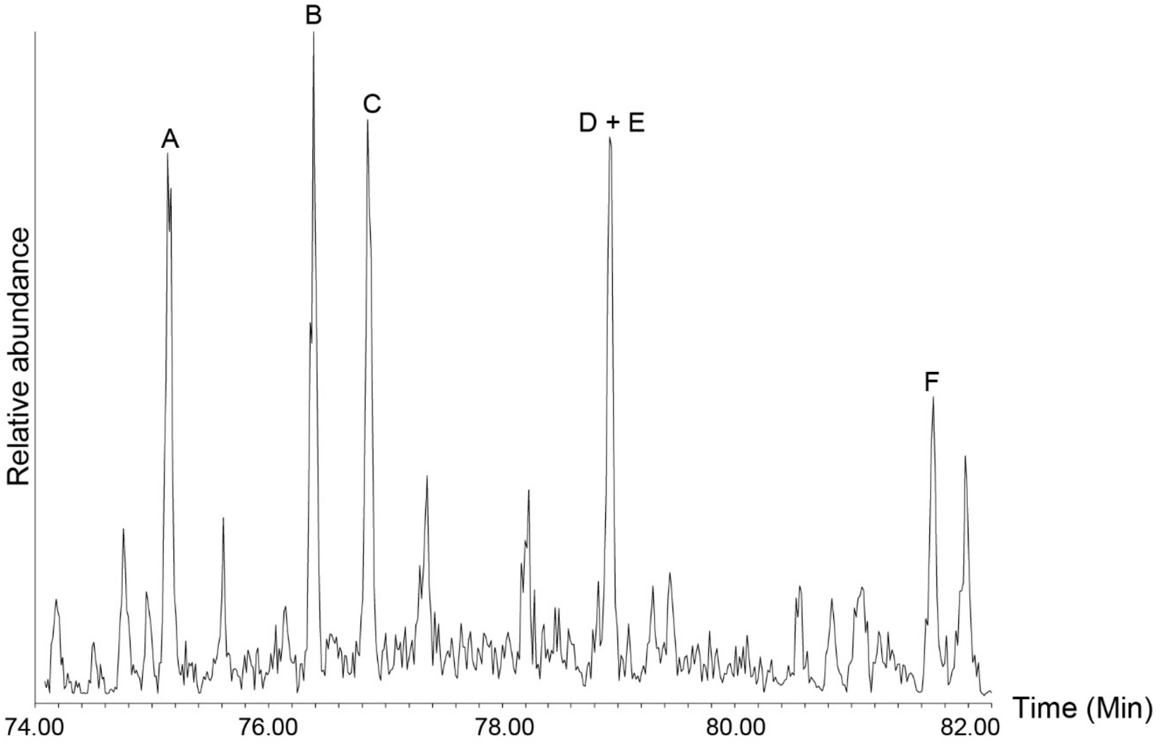

Figure S1: GC-MS *m/z* 257 chromatogram of aliphatic fraction showing presence of diasterenes. A: 10α, Δ13(17) diacholestene 20*S*; B: 10α, Δ13(17) diacholestene 20*R*; C: 10α, Δ13(17) 24-methyldiacholestene 20*S* (*Tentatively identified based on location of *R*-isomer and mass spectrum); D:10α, Δ13(17) 24-methyldiacholestene 20*R*; E: 10α, Δ13(17) 24-ethyldiacholestene 20*S*; F: 10α, Δ13(17) 24-ethyldiacholestene 20*R*. Compounds were tentatively identified based on comparison of retention time and peak patterns with sample where compounds had been previously identified [e.g. 26], confirmed using mass spectrum of peaks.

26. Melendez I, Grice K, Schwark L. Exceptional preservation of Palaeozoic steroids in a diagenetic continuum. Scientific Reports. 2013;3:2768.
